# Emergence of *Parafilaria bovicola* in Austria

**DOI:** 10.3390/ani11102966

**Published:** 2021-10-14

**Authors:** Alexandra Hund, Johannes Reithofer, Bita Shahi Barogh, Maria Sophia Unterköfler, Josef Harl, Hans-Peter Fuehrer

**Affiliations:** 1 Department of Farm Animals and Veterinary Public Health, University Clinic for Ruminants, University of Veterinary Medicine Vienna, 1210 Vienna, Austria; alexandra.hund@lazbw.bwl.de (A.H.); JohannesReithofer96@gmx.at (J.R.); 2 Agricultural Center for Cattle, Grassland, Dairy, Game and Fisheries of Baden-Württemberg (LAZBW), 88326 Aulendorf, Germany; 3 Institute of Parasitology, Department of Pathobiology, University of Veterinary Medicine Vienna, 1210 Vienna, Austria; Bita.ShahiBarogh@vetmeduni.ac.at (B.S.B.); Maria.Unterkoefler@vetmeduni.ac.at (M.S.U.); 4 Institute of Pathology, Department of Pathobiology, University of Veterinary Medicine Vienna, 1210 Vienna, Austria; Josef.Harl@vetmeduni.ac.at

**Keywords:** *Parafilaria bovicola*, cattle, parafilariosis

## Abstract

**Simple Summary:**

Bovine parafilariosis is a disease caused by the helminth *Parafilaria bovicola* (Filariidae, Nematoda). Flies transmit the parasite, which grows to adulthood in an unknown location in the affected animals. The adult female worms are located in nodules under the skin, which they penetrate and lay their eggs in the fluid leaking from the site. There is virtually no information about *Parafilaria bovicola* in Austria. In this study, these parasites were documented in the provinces of Lower Austria, Upper Austria, Styria, Salzburg, Carinthia, and Tyrol. With a high number of cases during the 2020 study period, it can be assumed that the number of reports will increase in the near future.

**Abstract:**

Veterinarians reported cases of cutaneous bleeding in cattle in Austria in the spring and summer of 2020. It was our goal to confirm the tentative diagnosis of parafilariosis by identifying *Parafilaria bovicola* in exudate samples using molecular methods for the first time in Austria. We asked veterinarians in the field to collect exudate from typical lesions on cattle. We performed polymerase chain reactions (PCRs) and sequenced a 674-bp section of the mitochondrial cytochrome oxidase subunit I in all positive samples. Overall, in 57 of 86 samples, *P. bovicola* was confirmed by PCR in cattle from Lower Austria, Upper Austria, Styria, Salzburg, Carinthia, and Tyrol. Sequencing detected four different haplotypes or genotypes, respectively, indicating multiple routes of introduction. We conclude that parafilariosis has spread in Austria and we expect that the number of reports of clinical signs and losses due to carcass damage will increase in the future.

## 1. Introduction

Bovine parafilariosis is a parasitic disease caused by the nematode *Parafilaria bovicola* that was first described by Tubangui [1] in the Philippines. Parafilariosis is characterized by the appearance of raised nodules on the neck and body of cattle, which may bleed profusely [2]. These nodules contain adult ovoviviparous females of *P. bovicola*, which penetrate the skin and release eggs and microfilariae (L1 larvae) into the serosanguinous fluid leaking from the site. The L1 larvae are ingested by *Musca* spp. (such as *M. autumnalis*, a species known to be endemic in Austria) and develop into infective L3 larvae, which are transmitted to cattle and cause cutaneous bleeding after a long period of prepatency of seven to ten months [3,4].

In Europe, Daslakow [5] identified a parasite he thought was identical to *P. bovicola* as described by Tubangui [1] at an abattoir in Sofia, Bulgaria in 1944. Tubangi had only described two female adult worms morphologically, whereas Daslakow found parafilariosis in 60 of 410 examined cattle. In these, he isolated up to 124 male and female *P. bovicola* specimens per animal. In 1948, cases of parafilariosis in Transylvania, an area that is in the center of present-day Romania, were reported. The author found the parasite in many locations and concluded that it must be widely spread in Romania already [6].

The disease was then described in Sweden in 1978 [7] and again in 2000 [8], where it is now regarded as endemic, but it was not found in Finland, the neighboring country [9]. Parafilariosis was first diagnosed in Belgium in 2009 [10] and was later found to be spreading in several Belgian provinces [11]. Single cases were described in Ireland in 1997 and in The Netherlands in 2007, both in bulls imported from France [12,13]. In both cases, the disease did not seem to spread any further. There are other reports of parafilariosis in Charolais cattle imported from France, for example in Canada [14,15]. In France, the disease seems to be present in the regions of Charolais and the southwest including Piemont Pyrénéen and Piemont du Massif Central, but has rarely been described [16,17]. Bech-Nielsen et al. [18] assumed that the parasite was of little economic concern and thus ignored in France.

Parafilariosis was first confirmed recently by microscopy of filariid eggs and parts of an adult worm retrieved by biopsy in two locations of Bosnia and Herzegovina [19]. The authors conducted a telephone survey with veterinarians in the possible endemic area but only three of 28 veterinarians had observed the symptoms in the past.

In Austria, the clinical symptom “spontaneous cutaneous hemorrhage” became of interest as a differential diagnosis to bovine neonatal pancytopenia [20]. Symptomatic cattle were first observed in the provinces of Carinthia, Styria, and Salzburg in 2009 and attributed to *P. bovicola* based on clinical signs and the epidemic situation [21]. *Parafilaria bovicola* has since been considered endemic in parts of Carinthia.

The route of introduction to Austria is unknown, but lesions typical of *P. bovicola* were described in the neighboring countries, in southwestern and southern Germany and later in Italy. In both countries, species identification of *P. bovicola* was based on morphological characteristics using microscopy only [22,23]. Molecular methods have since been established to identify nematodes on a species level using the mitochondrial gene cytochrome c oxidase 1 (*COI*); *COI* haplotypes can be used to studying population structure and genetic diversity [24,25].

It was the goal of our study to identify the cause of cases of cutaneous bleeding in cattle in new areas in Austria and to confirm our clinically tentative diagnosis of parafilariosis by identifying *P. bovicola* in exudate samples using molecular genetic methods. In all samples that were polymerase chain reaction (PCR) positive, we sequenced a 674 bp section of the mitochondrial cytochrome oxidase subunit I.

## 2. Materials and Methods

### 2.1. Sample Collection

Several cases of punctual bleeding from the skin of cattle were reported to the University Clinic for Ruminants at the University of Veterinary Medicine Vienna. We suspected parafilariosis, drafted an information letter together with a sample submission sheet and a questionnaire (Table 1), and distributed these to veterinarians in Austria via the Animal Health Services (Tiergesundheitsdienste) of the federal states. We asked veterinarians in the field to collect exudate from typical lesions on cattle using sample collection tubes and to freeze the samples at −20 °C. The samples were then collected by the medical logistics company medlog© and transported to the Institute of Parasitology at the University of Veterinary Medicine Vienna for further analysis. Some veterinarians were not able to collect samples themselves so J.R. collected samples on farms in Styria and Lower Austria.

### 2.2. Laboratory Analysis

DNA was extracted from the exudates using a DNeasy^®^ Blood and Tissue DNA extraction kit according to the manufacturer’s instructions (Qiagen, Hilden, Germany). Conventional PCRs, targeting a 674 bp section of the mitochondrial cytochrome c oxidase subunit I (*COI*) gene using the primers H14FilaCOIFw and H14FilaCOIRv, were performed as reported previously [26]. PCR products were separated by electrophoresis in 2% agarose gels stained with Midori Green Advance DNA stain (Nippon Genetics Europe, Düren, Germany). All positive PCR products were sequenced in both directions using Sanger sequencing at LGC Genomics GmbH, Berlin, Germany. The sequences were analyzed using Bioedit 7.5.0.3 [27]. The resulting sequences were compared for similarity to sequences available in GenBank (http://www.ncbi.nlm.nih.gov/BLAST) (accessed on 7 July 2021) and BOLD Systems (https://www.boldsystems.org/) (accessed on 7 July 2021). Moreover, sequences were uploaded to GenBank and BOLD Systems (accession numbers: MZ563376-MZ563429).

### 2.3. Data Analysis

A maximum likelihood tree was calculated for the *Parafilaria* sequences using the W-IQ-TREE web server (http://iqtree.cibiv.univie.ac.at/ (accessed on 7 July 2021) [28]) by applying the best-fit model TIM3+G4+F and performing 1000 bootstrap replicates. A sequence of *Dirofilaria repens* (MW590257) was used as the outgroup.

A median-joining haplotype network was calculated with Network 10.2.0.0 (Fluxus Technology Ltd., Suffolk, UK), applying the default settings. The network was graphically prepared and provided with information on the counties in Network Publisher v.2.1.2.3 (Fluxus Technology Ltd.) and finalized with Adobe Illustrator CC v.2015 (Adobe Inc., San José, CA, USA).

To illustrate the phylogenetic relationships of the genus *Parafilaria*, a maximum-likelihood tree was calculated with the *COI* sequences of other members of the order Spirurida. The sequences were obtained by blasting a *COI* sequence of *P. bovicola* against the Spirurida in the NCBI GenBank (accessed on 25 September 2021). The sequences were then aligned and sorted using the default option (FFT-NS-2) in MAFFT v.7.311 [29]. Since most sequences did not cover the 674 bp section analyzed in the present study, the alignment was trimmed to 576 bp. All sequences featuring obvious sequencing errors and ambiguity characters were removed from the alignment and the sequences were collapsed to haplotypes using DAMBE v. 7.0.5.1 [30]. To reduce the size of the alignment, only two sequences were kept per species, resulting in 239 haplotypes. A sequence of *Ascaris suum* (KY045800) was used as the outgroup. The tree was calculated using the W-IQ-TREE web server (http://iqtree.cibiv.univie.ac.at/) (accessed on 25 September 2021) by applying the best-fit model TIM3+G4+F and performing 1000 bootstrap replicates. The sequence alignment is provided in Supplementary File S1.

## 3. Results

**Figure 1:**
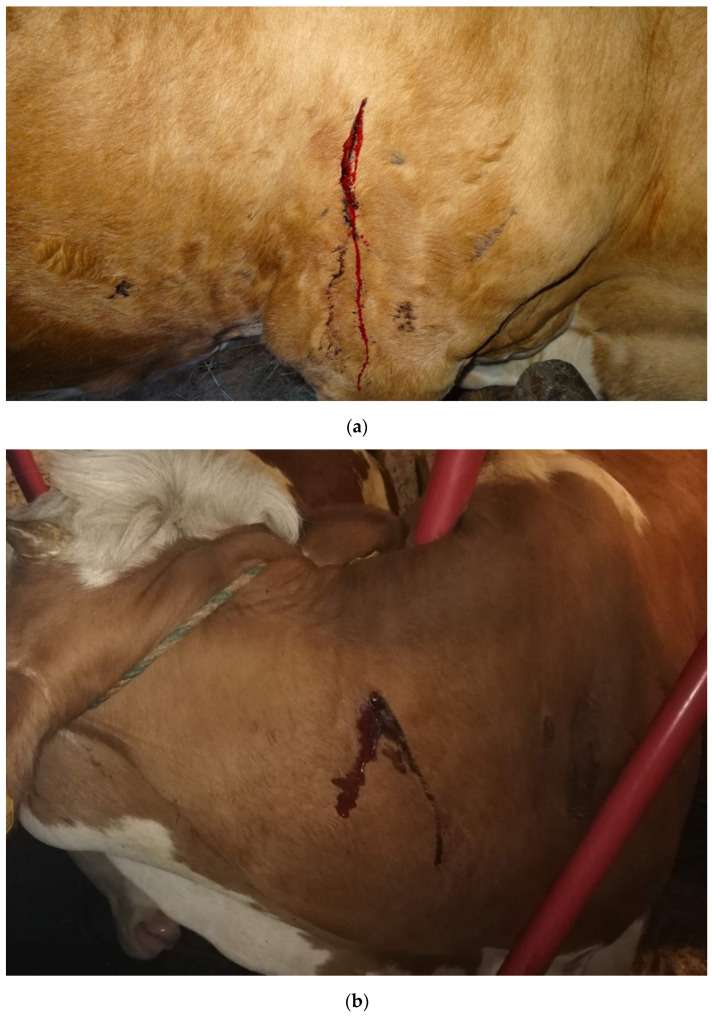
Cattle affected by *P. bovicola* with bleeding spots on (**a**) the thorax in the shoulder region and (**b**) the neck. Photo credit: Susanne Möser (**a**), Johannes Reithofer (**b**).

Photographs of affected cattle provided by veterinarians and farmers showed punctual bleeding in cattle with dried and fresh bloody streaks of exudate in the typical areas of the dorsal aspect of the body including the head, neck, shoulders, withers, dorsal part of the ribs, and the gluteal region. Examples can be seen in Figure 1.

**Figure 2:**
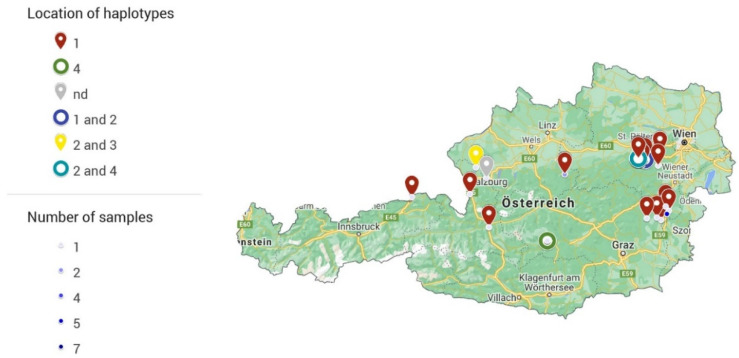
Location of haplotypes and number of positive samples submitted by town. Please visit https://www.google.com/maps/d/edit?mid=1zjFBQwKYfcNieR0q0vtQLkYcYkbKObSy&usp=sharing (accessed on 20 August 2021) for an interactive map.

**Figure 3:**
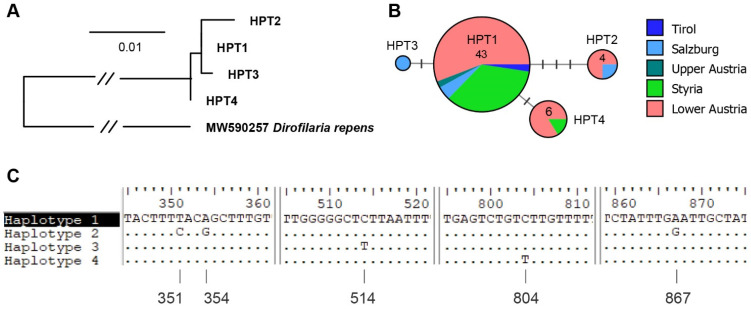
(**A**) Maximum likelihood tree of the four cytochrome c oxidase subunit I (*COI*) haplotypes (HPT1–4) with a *Dirofilaria repens* sequence from GenBank (accession number: MW590257) as the outgroup; (**B**) median-joining haplotype network showing the distribution of different haplotypes per federal state and the number of individuals; (**C**) sequence alignment showing the nucleotide positions (counting from the 5′-end of the *COI*) differing between the four *COI* haplotypes.

Overall, 86 samples from 62 cattle from Lower Austria, Upper Austria, Styria, Salzburg, Carinthia, and Tyrol were submitted to the Institute of Parasitology of the University of Veterinary Medicine Vienna (Table 2). In 57 of these 86 samples (*n* = 41 animals), *P. bovicola* was confirmed by PCR and sequencing. One sample did not contain enough exudate for the test to be performed. If multiple samples from the same animal were submitted, the results of the PCRs were consistent in all animals, except for one case, meaning that *P. bovicola* DNA was detected either in all samples or none. In one animal, two of three samples were positive. All but three samples from three different animals featured sequences of high quality and could be assigned to one of four haplotypes (haplotype 1: GenBank accession number MZ563421, haplotype 2: MZ563418, haplotype 3: MZ563406, haplotype 4: MZ563380). Interestingly, in four animals, two different haplotypes were identified in different samples (haplotype 1 and 2 (2x), haplotype 2 and 4, and haplotype 2 and 3). A map showing the geographic distribution of sampling locations and haplotypes is provided in Figure 2. The haplotypes showed a close resemblance, differing by 1–4 bp from each other. A Maximum likelihood tree, DNA haplotype network and an alignment showing the nucleotide differences in the *COI* between *P. bovicola* haplotypes is provided in Figure 3. A maximum-likelihood tree was calculated with the *COI* sequences of *P. bovicola* and other members of the order Spirurida (Supplementary File S2). The genus clades were mostly well-supported, but the deeper nodes in the tree obtained only low bootstrap values. Based on the 576 bp *COI* section, *Parafilaria* is closest related to *Thelazia* and the two genera cluster in a clade with maximum support.

Most samples were collected in June and July, with only one and two negative samples collected in August and September, respectively. Of the 37 samples collected in June, 19 were positive, and of the 28 samples collected in July, 27 samples were positive for *P. bovicola*.

Most samples originated from animals kept on dairy farms (53 individuals from 34 farms) and only samples of seven animals came from suckler cow herds on five different farms. No further information or history was submitted with the samples from two animals. The farms kept between 2 and 95 cattle (mean: 43.5, median: 35 cattle) and the farmers reported that between 1 and 8 cattle (mean 3.3, median 2.5) were or had been affected by bleeding from the skin typical of *P. bovicola*. The animals were between 7 months and 10 years old, with a mean age of 52 months and a median age of 47 months. Only two animals were male.

Of those studied, 54 animals had access to pasture or an outdoor pen. Of the eight animals where access to the outdoors was unknown or not given, only one animal in each category was positive for *P. bovicola.* Eight farmers reported that they had observed the symptoms from as early as six years ago up until early 2020, the year of sample collection. However, *P. bovicola* was only detected in cattle from three of those farms. At the farms where 32 of the animals were kept, fly control was conducted using adhesive or insect electrocutor traps or using pour-on formulations containing pyrethroids. On farms where 34 of the animals were kept, the cattle received a regular anti-parasitic treatment using macrocyclic lactones. However, even though preventive measures were taken, in 26 of 32 animals from farms where fly control was applied and in 28 of 34 animals from farms where the cattle were dewormed, *P. bovicola* was detected. In 21 of 24 animals from farms where both fly control and deworming were performed, *P. bovicola* DNA was detected.

Loss of production or abnormal behavior in relation to the occurrence of parafilariosis were only reported at one farm where the somatic cell count of the cattle had increased. Two farms reported no information, and when samples were sent from the others, nothing was noted. One farmer reported that the affected animal had aborted a calf six weeks before the calculated calving date but did not attribute this to the skin bleeding. No *P. bovicola* could be detected in the sample of this particular animal.

## 4. Discussion

*Parafilaria bovicola* was present in 25 cattle herds in five states of Austria. Based on the questionnaire sent out, participants collected samples from cattle showing the typical symptoms of parafilariosis, namely, localized bleeding from the skin. We assume that most cattle showing these symptoms were affected by *P. bovicola*. Though trauma or insect bites could cause the same symptoms [10], we suspect that, instead, a lack of genetic material in some samples was likely caused due to a suboptimal sampling technique or timing, or high temperatures during storage or shipping. The fact that one veterinary practice submitted nine negative samples from five farms collected in June supports our proposal. Only negative samples were submitted in August and September, indicating that there may be differences in the presence of eggs and/or larvae through the season. This may lead to a lower number of positive samples by the end of the season but should not have had an effect on samples collected in June.

We did not obtain information on the breed of the affected animals. However, in Austria, about 75% of the cattle population consists of *Fleckvieh*. Breeds like Charolais or Blonde d’Acquitane that introduced parafilariosis from France to countries like Canada or Belgium [12,13,14,15] only make up about 1% of cattle in Austria [31]. However, it is not impossible that breeders introduced the parasitosis by purchasing subclinically affected breeding stock from endemic regions like in the case of *Besnoitiosis* in Switzerland and Germany [32,33].

Sequencing resulted in the detection of four haplotypes. Only one entry of *P. bovicola* was available on GenBank (accession number: MG983751) for comparison [34], which showed 100% identity to haplotype 1 with a query coverage of only 96%. Therefore, the sample was not included in the analysis.

Three different haplotypes were detected in both Lower Austria and Salzburg. Together with reports of parafilariosis in several neighboring countries [22,23,34], this leads to the conclusion that it is unlikely that the infections originated from a point source, but rather from different routes of introduction. The first suspected cases of parafilariosis—which were not confirmed using molecular methods—were reported in Austria over a decade ago and the disease is considered endemic in parts of Carinthia [21]. Likely, cattle in other parts of Austria have displayed symptoms before, but we suspect a surge of clinical cases in 2020, which might have exceeded the threshold required to be noted as unusual.

The true extent of the problem, the epidemiological situation in Austria, is unknown because our study is based on a convenience sample and we relied on the voluntary participation of veterinarians and farmers. However, we are convinced that most Austrian veterinarians received our information letter distributed by the Animal Health Services in all federal states, meaning that everyone who was interested had a chance to participate.

Lesions were usually observed between December and July in the northern hemisphere [2,3]. Even though the call to submit samples was sent out in June, which is late in the typical “bleeding season”, we received a substantial number of samples from five federal states. Therefore, we conclude that *P. bovicola* has spread in Austria and is most likely endemic in most parts of the country. Many farms in Austria are not closed operations, meaning that farmers buy animals at cattle markets or directly from other farms. This livestock movement allows for the distribution of asymptomatic animals that carry *P. bovicola* larvae. Once these animals start showing symptoms, the reproductive cycle of the parasite can be completed because the vector flies are ubiquitous [35]. Thus, the parasitic disease can spread in the new herd. Bech-Nielsen et al. [18] observed an expansion of the endemic *Parafilaria* area in Sweden of about 50 km/year through airborne transport by vector flies and the movement of these flies and cattle via transport vehicles.

Responses from the questionnaire indicate that many cattle were affected by parafilariosis on farms where fly control was performed and cattle were regularly treated with macrocyclic lactones. Unfortunately, antiparasitic treatment is ineffective against early larval stages. Hence, the metaphylactic treatment of animals from affected herds is useless. Symptomatic animals that have been treated show a rapid resolution of lesions [36,37] but may start bleeding again after only a few weeks [13].

The finding that farmers noted little to no effect of *P. bovicola* on the condition of affected animals are in accordance with previous reports [3,19]. The main cause for economic losses associated with *P. bovicola* is the carcass quality. The parasite causes edematous changes that may turn the form yellow to greenish, covering an area of 490.7 cm^2^ on average, leading to the condemnation of 1.23 up to 6 kg of trimmings, especially in young bulls [4,38]. Most lesions are superficial but extensive involvement of the muscles are found in more severe cases [39,40]. Superficial lesions may be mistaken for contusions that occurred during handling or transport [4] and were, therefore, not reported in our study.

## 5. Conclusions

With a substantial number of positive samples from all over Austria, we conclude that *P. bovicola* has spread and will become endemic in the country in the near future if this is not the case already. We expect that reports of symptoms and lesions will occur more frequently as veterinarians and farmers become increasingly aware of the disease. It would be beneficial to implement a voluntary surveillance program where farmers and veterinarians submit samples to gain a better understanding of the true situation in Austria and other European countries. As the voluntary participation in our study was taken up well, we would expect such a program to yield valuable results and help us to understand the mode and pace of the spread of *P. bovicola* in different climatic zones and landscapes.

## Figures and Tables

**Table 1:** History questions for farmers as distributed through veterinarians.

History Question
What is the type of farm: dairy, suckler cow, or fattening? How many cattle are housed at the affected farm? How many cattle show the symptoms typical of parafilariosis (skin bleeding)? Did you observe any changes in behavior or a decrease in production in the affected cattle? If yes, please describe. Do the affected animals have access to pasture or an outdoor pen? Have you observed the symptoms in the past? If yes, since when? Are you using fly control on the farm? If yes, what do you use? Do you deworm the animals on your farm? If yes, what do you use? Have you submitted samples of the bleeding lesions? If yes, what was the result?

**Table 2:** Overview of the number of samples, affected cattle, herds, and haplotypes detected.

Federal State	Samples Subm.	Animals Subm.	Herds Subm.	Samples Positive	Animals Positive	Herds Positive	Haplotypes
Lower Austria	34	23	9	32	22	8	1, 2, 4
Styria	23	15	13	18	12	11	1, 4
Upper Austria	11	8	7	2	2	2	1
Salzburg	13	11	7	4	3	3	1, 2, 3
Carinthia	3	3	3	0	0	0	n.d.
Tyrol	2	2	2	1	1	1	1
Total	86	62	41	57	40	25	

n.d., not detected; subm., submitted.

## Supplementary Materials

The following are available online at https://www.mdpi.com/article/10.3390/ani11102966/s1, File S1: COI sequence alignment, species names and accession numbers for the maximum likelihood tree, File S2: Maximum likelihood tree (1000 replicates) featuring COI sequences (576 bp) of *Parafilaria bovicola* and other members of the suborder Spirurida.
