# Supplementary material for: Emergence of Parafilaria bovicola in Austria
Source: Animals (Basel). 2021 Oct 14;11(10):2966. doi: 10.3390/ani11102966 (PMC8532764; doi:10.3390/ani11102966)

Supporting File 2 Maximum likelihood tree (1000 replicates) featuring *COI* sequences (576 bp) of *Parafilaria bovicola* and other members of the suborder Spirurida. Ascaris suum (KY045800) was used as outgroup. Maximum likelihood boostrap values are indicated at all nodes. The genus clades were collapsed. An alignment featuring the species names and accession numbers is provided in Supporting File 1.

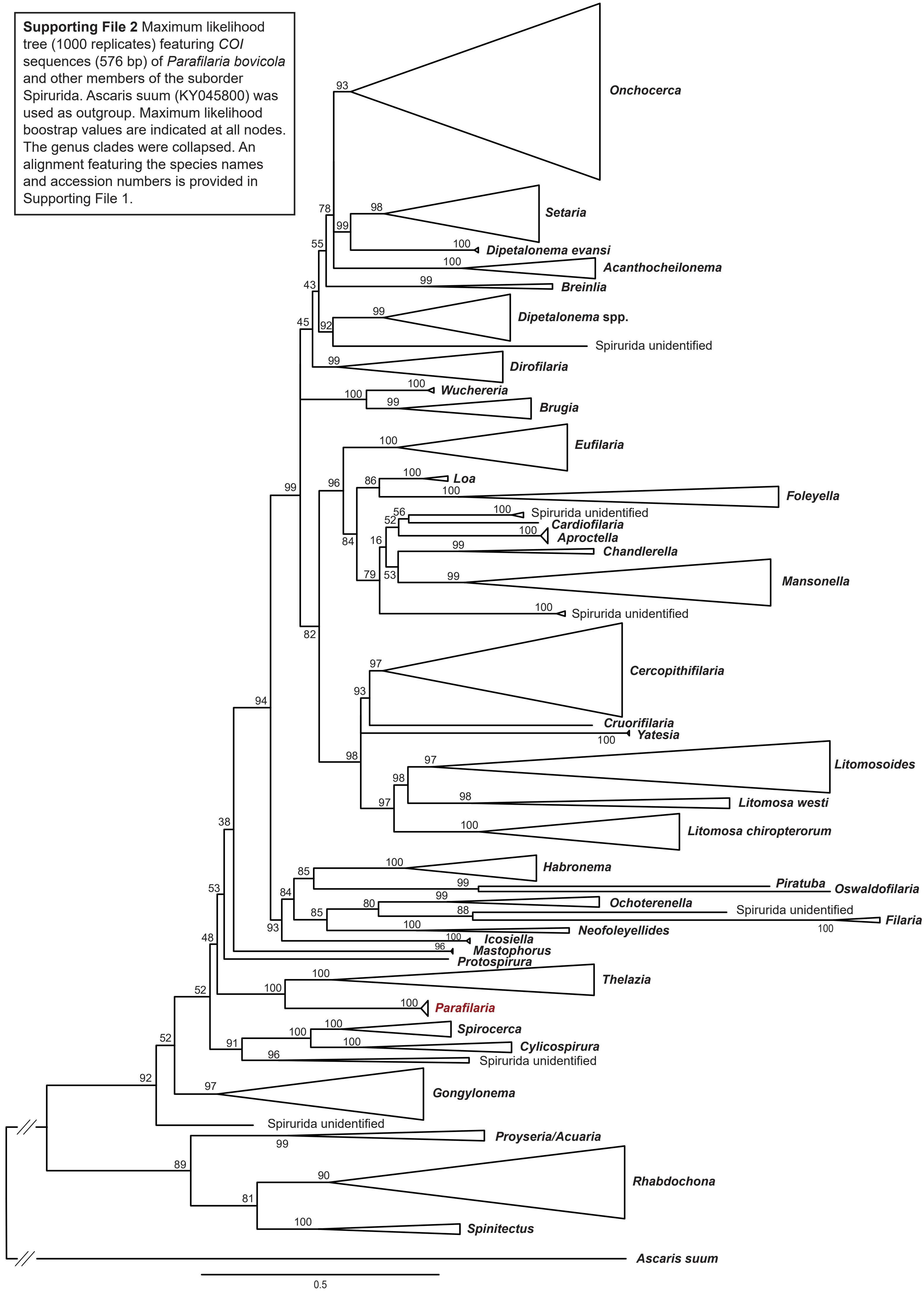

Supplement: Supplementary file 1 [file animals-11-02966-s001.zip › Supporting File S2.pdf]
